# Dermatological and gastrointestinal adverse reactions in ocrelizumab treated patients with multiple sclerosis: a case series

**DOI:** 10.3389/fimmu.2026.1753387

**Published:** 2026-03-09

**Authors:** Lena K. Höpner, Ella Marschall, Patrick Schindler, Vilmar Frauendorf, Michael Böhmig, Florian Rakers, Diana Karimi, Claudius Faber, Klemens Ruprecht, Carolin Otto

**Affiliations:** 1 Department of Neurology, Charité - Universitätsmedizin Berlin, corporate member of Freie Universität Berlin and Humboldt-Universität zu Berlin, Berlin, Germany; 2 Department of Gastroenterology, MEOCLINIC GmbH, Berlin, Germany; 3 Dr. Spitz & Kollegen, Practice For Gastroenterology, Berlin, Germany; 4 Department of Neurology, Universitätsklinikum Jena, Jena, Germany; 5 Pathologie München-Nord, practice for pathology, Munich, Germany; 6 Division of Neuroimmunology, Department of Neurology, University of Heidelberg, Heidelberg, Germany

**Keywords:** anti-CD20 therapy, colitis, dermatological adverse reactions, gastrointestinal adverse reactions, lichen planus, multiple sclerosis, ocrelizumab, psoriasis

## Abstract

**Background:**

Anti-CD20 therapies are widely used in patients with multiple sclerosis (pwMS). Recognition of rare adverse reactions to these therapies is therefore important.

**Objectives:**

To report dermatological and gastrointestinal adverse reactions in a single-center cohort of ocrelizumab treated pwMS.

**Methods:**

Retrospective analysis conducted at a multiple sclerosis outpatient clinic of Charité – Universitätsmedizin Berlin, Berlin, Germany, between March 2020 and February 2025.

**Results:**

Among 447 ocrelizumab treated pwMS, 8 (1.8%) developed dermatological adverse reactions after a median (range) of 20.5 (6-72) months following start of therapy, including lichen planus (n=2), rosacea (n=1), psoriatic arthritis (n=1), guttate psoriasis (n=1), psoriasis vulgaris (n=1), nail psoriasis (n=1) and palmoplantar psoriasis (n=1). Another 5 (1.1%) patients developed gastrointestinal adverse reactions 24 (0.25-77) months after starting therapy, including Crohn’s disease (n=1), toxic colitis (n=1), lymphocytic colitis (n=1), perforated appendicitis (n=1) and acute cholecystitis (n=1). Due to these adverse reactions, ocrelizumab was stopped in 7/13 patients. At last follow-up, adverse reactions had completely improved in 4/13, incompletely improved in 6/13, and persisted in 3/13 patients.

**Conclusions:**

Clinicians should be aware of dermatological and gastrointestinal adverse reactions associated with ocrelizumab, which can develop from a few weeks up to six years after start of therapy.

## 1 Introduction

Recombinant monoclonal antibodies targeting the CD20 molecule expressed on B cells (anti-CD20 therapies) have proven to be effective therapies for multiple sclerosis (MS) (1–6). Anti-CD20 therapies deplete circulating B cells, but the precise mechanisms underlying their beneficial effects in patients with MS (pwMS) are not completely understood (7). Currently available anti-CD20 therapies for MS comprise rituximab, ocrelizumab, ofatumumab, and ublituximab (7). Due to their increasing usage in pwMS, recognition not only of common, but also of rare adverse reactions to anti-CD20 therapies is of high clinical relevance.

The most frequent adverse reactions to anti-CD20 therapies are infections of the upper respiratory and urinary tract and infusion-related reactions (1, 2). Furthermore, in recent years, dermatological adverse reactions, in particular psoriasis, have been reported in patients treated with anti-CD20 therapies (8, 9). Likewise, gastrointestinal adverse reactions, such as inflammatory bowel diseases (IBD), including acute colitis and chronic inflammatory bowel diseases (CID), have been recognized (10, 11). This resulted in a safety-related label change by the Food and Drug Administration (FDA) concerning colitis as a potential complication of therapy with ocrelizumab in April 2022 (12).

To further raise awareness for rare but potentially serious adverse reactions to anti-CD20 therapies, we here report dermatological and gastrointestinal adverse reactions in a large single-center cohort of 447 ocrelizumab treated pwMS.

## 2 Patients and methods

### 2.1 Patients

PwMS included in this study were treated at the MS outpatient clinic, Charité Campus Mitte, Charité - Universitätsmedizin Berlin, Berlin, Germany, between March 5, 2020, and February 13, 2025. Inclusion criteria were age ≥18 years, a diagnosis of MS according to the McDonald 2017 criteria (13) made by neurologists specialized in MS, and treatment with at least 300 mg ocrelizumab. The ocrelizumab treatment regime typically consisted of two infusions of 300 mg administered intravenously two weeks apart, followed by administration of 600 mg ocrelizumab every 6 months. On an individual basis, treatment intervals were extended to more than 6 months in some of the patients. In few cases, pwMS had been treated with rituximab, administered at a dose of 1000 mg intravenously two weeks apart and followed by six-monthly infusions of 1000 mg, prior to start of therapy with ocrelizumab. In these instances, the day on which rituximab was first administered was considered the start of anti-CD20 therapy. Patients treated with ofatumumab and ublituximab were not included in the analysis. Patients under 18 years of age were excluded, too.

Following start of ocrelizumab therapy, patients were typically seen every six months at the MS outpatient clinic. During all such outpatient visits, patients were asked whether they had developed new symptoms and/or potential adverse reactions under treatment with ocrelizumab. Patients who reported gastrointestinal symptoms, such as abdominal pain or diarrhea, were referred to specialists in gastroenterology for further diagnostics, e.g. ultrasound or colonoscopy. If a diagnosis of a gastrointestinal disease was confirmed by a specialist in gastroenterology, the patient was considered to have a potential gastrointestinal adverse reaction to ocrelizumab and included in this case series. Patients who reported skin changes while on treatment with ocrelizumab were referred for dermatological evaluation. If a diagnosis of a dermatological disease was confirmed by a specialist in dermatology, the patient was considered to have a potential dermatological adverse reaction and included in this case series.

The outcome of dermatological and gastrointestinal adverse reactions was categorized into complete improvement (resolution of symptoms), incomplete improvement (partial resolution of symptoms), and no improvement (persistent symptoms) at the last follow-up examination.

### 2.2 Statistics

Statistical analyses were performed using GraphPad Prism 10.06.0. For descriptive statistics, quantitative data are presented as median and absolute range, whereas categorical data are displayed as absolute (n) and relative (%) frequencies. As both groups (dermatological and gastrointestinal adverse reactions) were unpaired and nonparametric, the Mann-Whitney-U-Test was used for group comparison. To analyze statistical significance of differences in frequencies, two-tailed Fisher’s exact test was applied. *P*-values <0.05 were considered statistically significant.

### 2.3 Ethical approval and patient consent

The study was approved by the ethical committee of Charité - Universitätsmedizin Berlin (EA1/074/25). Written informed consent was obtained from all reported patients in this study, and additional written informed consent was obtained from all three patients of whom image material is included in this publication.

## 3 Results

### 3.1 Patients

Between March 5, 2020, and February 13, 2025, 447 pwMS were treated with at least 300 mg ocrelizumab at the MS outpatient clinic, Charité Campus Mitte, Charité - Universitätsmedizin Berlin, Berlin, Germany. Among them, some patients had previously already been treated with intravenous rituximab (1000 mg) in six-monthly intervals.

Altogether, 8/447 (1.8%) pwMS developed a dermatologically confirmed dermatological disorder and another 5/447 (1.1%) a gastroenterologically confirmed gastrointestinal disorder under therapy with ocrelizumab during the observation period. These dermatological and gastrointestinal disorders were considered potential adverse reactions to ocrelizumab and are further described below.

### 3.2 Dermatological adverse reactions

Demographic and clinical characteristics of pwMS (5 women, 3 men) who developed dermatological disorders following start of ocrelizumab therapy are summarized in **Table 1**. The patients’ median (range) age at onset of dermatological symptoms was 41.5 (27-58) years. Dermatological symptoms developed after a median ocrelizumab treatment duration of 20.5 (6-72) months. At symptom onset, pwMS with dermatological disorders had received a cumulative ocrelizumab dose of 2100 (600-4200) mg during a median of 3.5 (1-7) infusions. The median follow-up period after onset of dermatological symptoms was 25.5 (9-77) months. Five of the eight patients had been treated with at least one disease modifying therapy, including glatiramer acetate (n=3), interferons (n=3), dimethyl fumarate (n=1), fingolimod (n=1), natalizumab (n=1) and mitoxantrone (n=1), prior to the start of therapy with ocrelizumab. Additionally, two patients had been treated with rituximab prior to the start of therapy with ocrelizumab.

**Table 1:** Demographic and clinical data of eight patients with MS who developed dermatological disorders under ocrelizumab therapy.

Patient #, gender (f/m), age (years)	MS type	Dermatological diagnosis	Biopsy proven	Interval between start of anti-CD20 therapy and onset of dermato-logical symptoms (months)	Cumulative dose of anti-CD20 therapy at onset of dermatological symptoms (mg)	Previous disease modifying treatments	Dermatological treatment	Adaptation of ocrelizumab therapy after onset of dermato-logical symptoms	Dermato-logical outcome	Follow-up period** (months)
1, f, 29	RRMS	Guttate psoriasis	Yes	23	3000 (Ocrelizumab)	Glatiramer acetate	Topical steroids	Continued	Complete improvement	23
2, m, 45	RRMS	Psoriasis vulgaris	Yes	18	1800 (Ocrelizumab)	None	Topical treatment (keeping moist)	Continued with 12 months treatment intervals	Incomplete improvement	51
3, f, 53	RRMS	Lichen planus mucosae, including vaginal, Lichen planus planopillaris	Yes	13	1800 (Ocrelizumab)	Interferon, Mitoxantrone, Glatiramer acetate, Natalizumab, Fingolimod	Topical and systemic steroids	Stopped	No improvement	12
4, f, 40	RRMS	Lichen planus and sclerosus vaginal	No	18	2400 (Ocrelizumab)	None	Topical steroids	Continued	No improvement	28
5^#^, m, 58	RRMS	Nail psoriasis	Yes	72	3600 (Ocrelizumab), 4000 (Rituximab)	Dimethyl fumarate	Topical steroids	Stopped*	Incomplete improvement	28
6, f, 43	RRMS	Psoriatic arthritis	No	52	4200 (Ocrelizumab)	Glatiramer acetate, Interferon,	First Upadacitinib then switch to Methotrexat	Stopped	Incomplete improvement	16
7^#^, m, 37	RRMS	Rosacea	No	72	600 (Ocrelizumab), 5000 (Rituximab)	None	Doxycycline and topical ivermectin	Continued with 12 months treatment intervals	Incomplete improvement	77
8, f, 27	RRMS	Palmoplantar psoriasis	No	6	1200 (Ocrelizumab)	Interferons	Topical steroids, 20% salicylic acid in petrolatum, protopic 0.1% ointment	Stopped, switch to cladribine	Complete improvement	9

f, female; m, male; MS, multiple sclerosis; RRMS, relapse-remitting multiple sclerosis.

*Anti-CD20 treatment was stopped due to severe COVID-19 pneumonia 22 months after onset of dermatological symptoms.

**The follow-up period was defined as the time from symptom onset until the last visit.

^#^These patients initially received rituximab, before treatment was switched to ocrelizumab in 2018. The start of anti-CD20 therapy was defined as the day of the first rituximab treatment.

In the following, we present the different dermatological disorders, their treatment, and subsequent adaptation of anti-CD20 therapy in detail.

#### 3.2.1 Psoriasis

Psoriasis was the most frequent dermatological complication, which occurred in five patients and included guttate psoriasis (n=1), psoriasis vulgaris (n=1), nail psoriasis (n=1), psoriatic arthritis (n=1) and palmoplantar psoriasis (n=1). Diagnosis was biopsy proven in 3/5 patients, confirmed by sonography in the patient with psoriatic arthritis and was based on clinical findings in the patient with palmoplantar psoriasis.

Except for the patient with psoriasis arthritis, topical steroids were used as the first-line dermatological treatment in patients with psoriasis. At the last follow-up visit, psoriasis had either completely (n=2) or incompletely (n=3) improved.

In detail, the patient with guttate psoriasis (patient #1) fully recovered under topical steroids and ocrelizumab treatment was continued without recurrence of symptoms until the last follow-up 23 months after symptom onset.

Topical treatment was associated with incomplete improvement at 51 months of follow-up in the patient with psoriasis vulgaris (patient #2) and ocrelizumab infusion intervals were extended to 12 months.

Nail psoriasis (patient #5) was treated with topical steroids, which was associated with incomplete improvement of symptoms at 28 months of follow-up. While ocrelizumab treatment was initially continued, it was stopped 22 months after the onset of dermatological symptoms, due to severe SARS-CoV-2 pneumonia.

Dermatological treatment of psoriatic arthritis (patient #6) was initiated with the JAK1-inhibitor upadacitinib. However, due to an insufficient response to upadacitinib the patient was switched to methotrexate, which was associated with a noticeable, albeit incomplete, reduction in symptoms at 16 months of follow-up. Ocrelizumab was discontinued in this patient.

**Figure 1:**
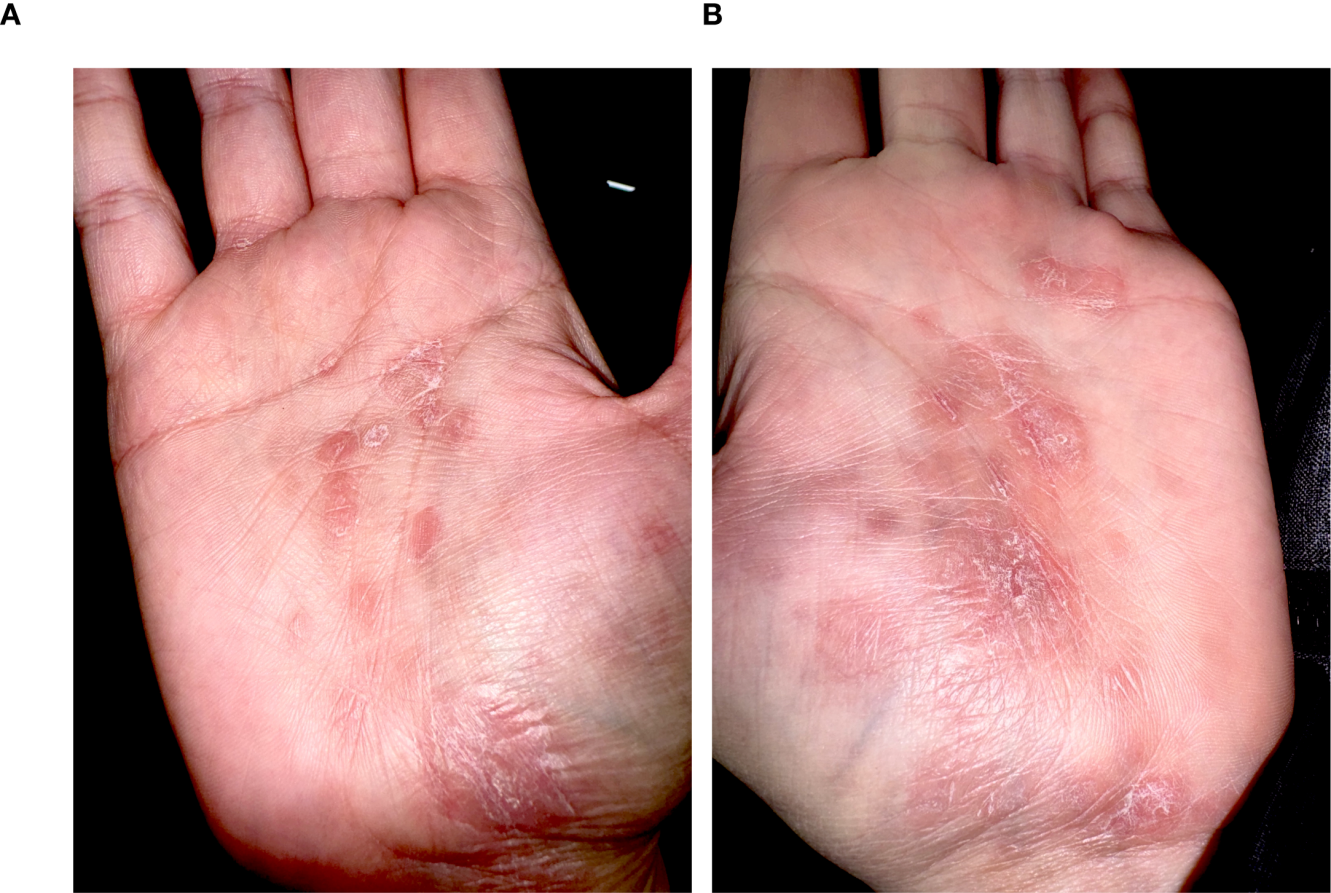
Palmar surfaces of a patient (patient #8) who developed psoriasis palmoplantaris following the first two infusions of ocrelizumab. Pictures **(A, B)** depict well-demarcated, erythematous, and hyperkeratotic plaques affecting both palms, without evidence of vesiculation or pustulation.

**Figure 1** shows the palmar surfaces of the patient with palmoplantar psoriasis (patient #8). In this patient, dermatological symptoms improved after local steroid therapy but worsened after a subsequent ocrelizumab administration. Ocrelizumab was consequently stopped and replaced by cladribine. At last follow-up, 9 months after onset of dermatological symptoms, the patient had a complete improvement of dermatological symptoms.

#### 3.2.2 Lichen planus

We also identified two patients with lichen planus. One of these patients (patient #3), a 53-year-old woman with a biopsy proven diagnosis, has previously been reported in detail (14). The patient had a history of previous disease modifying therapy (DMT) usage, including interferons, mitoxantrone, glatiramer acetate, natalizumab and fingolimod, with a therapy-free interval of six years between the last DMT and start of ocrelizumab. Lichen planus manifested clinically after three infusions of ocrelizumab and was severe, despite of treatment with topical and systemic steroids and cessation of ocrelizumab therapy. At the last follow-up, 12 months after symptom onset, the patient had no significant improvement of the oral mucosal affection.

The second patient (patient #4) was diagnosed with lichen planus based on typical clinical findings. Dermatological treatment consisted of topical steroids. Lichen planus did not cause any discomfort in this patient and anti-CD20 therapy was continued. The patient had no improvement of dermatological symptoms at 28 months of follow-up.

#### 3.2.3 Rosacea

Another patient (patient #7), who had previously been treated with a cumulative dose of 5000 mg of rituximab, developed rosacea one month after switching to ocrelizumab (cumulative dose 600 mg). Diagnosis was based on typical clinical findings and dermatological treatment included temporary doxycycline and topical ivermectin. Ocrelizumab treatment was continued in 12-month dosing intervals. The patient had an incomplete improvement of dermatological symptoms at last follow-up 77 months after onset of dermatological symptoms.

### 3.3 Gastrointestinal adverse reactions

Demographic and clinical characteristics of five female pwMS with gastrointestinal disorders under ocrelizumab therapy are summarized in **Table 2**. The median (range) age of the patients who developed gastrointestinal adverse reactions was 35 (25-55) years. The median treatment duration with ocrelizumab was 24 (0.25-77) months. At onset of gastrointestinal symptoms, patients had received a cumulative ocrelizumab dose of 2400 (300-7200) mg administered over a median of 4 (0.5-12) infusions. The median follow-up period after onset of gastrointestinal symptoms was 28 (9-49) months. 4/5 patients had previously been treated with at least one previous DMT, including glatiramer acetate (n=2), interferons (n=1), dimethyl fumarate (n=3) and fingolimod (n=2).

**Table 2:** Demographic and clinical data of five patients with MS who developed gastrointestinal disorders under ocrelizumab therapy.

Patient #, gender (f/m), age (years)	MS type	Gastro-enterological diagnosis	Biopsy proven	Interval between start of anti-CD20 therapy and onset of gastrointestinal symptoms (months)	Cumulative dose of anti-CD20 at onset of gastro-intestinal symptoms (mg)	Previous disease modifying treatments	Gastro-enterological treatment	Adaptation of ocrelizumab therapy after onset of gastrointestinal symptoms	Gastro-enterological outcome	Follow-up period (months)**
9, f, 25	RRMS	Crohn’s disease	Yes	24	2400 (Ocrelizumab)	Dimethyl fumarate, Glatiramer acetate	Topical and systemic steroids, Ozanimod	Stopped, switch to Ozanimod	Incomplete improvement	28
10, f, 47	RRMS	Toxic colitis	Yes	36	4200 (Ocrelizumab)	Dimethyl fumarate	Systemic steroids	Stopped	Incomplete improvement	9
11, f, 35	RRMS	Perforated appendicitis	Yes	1	600 (Ocrelizumab)	None	Surgery	Continued	Complete improvement	38
12, f, 34	RRMS	Cholecystitis	Yes	0.25	300 (Ocrelizumab)	Glatiramer acetate, Fingolimod	Surgery	Continued	Complete improvement	49
13, f, 55	RRMS	Lymphocytic colitis	Yes	77	7200 (Ocrelizumab)	Interferon, Fingolimod, Dimethyl fumarate	Systemic steroids	Stopped	No improvement	12

f, female; m, male; MS, multiple sclerosis; RRMS, relapse-remitting multiple sclerosis.

**The follow-up period was defined as the time from symptom onset until the last visit.

Details of the observed gastrointestinal disorders, their gastroenterological treatments and subsequent adaptations of anti-CD20 therapy are given below.

#### 3.3.1 Inflammatory bowel diseases

**Figure 2:**
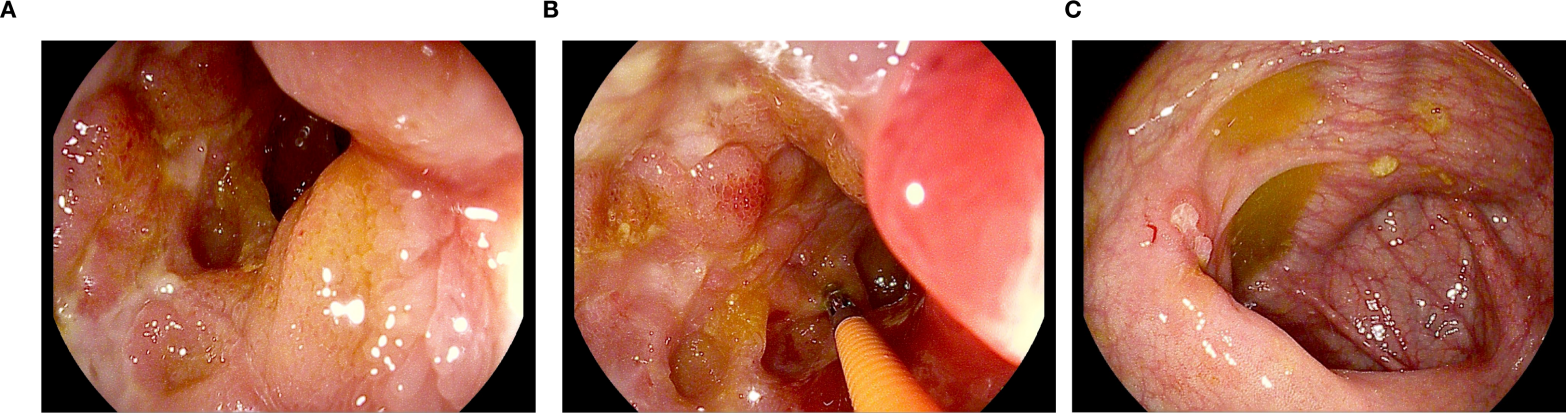
Endoscopic images of the ileum in a patient (patient #9) with Crohn’s disease, obtained 24 months after the initial administration of ocrelizumab. Pictures **(a, b)** demonstrate terminal ileitis, characterized by inflamed and edematous mucosal areas with ulcerations. **(c)** reveals fibrin-covered mucosal defects on the ileocecal valve.

Crohn’s disease was histologically confirmed in one woman (patient #9) with diarrhea and stomach pain who simultaneously developed skin lesions on the anterior tibia and in both auditory canals, which were attributed to extraintestinal manifestations of Crohn’s disease. Gastroenterological treatment consisted of topical and systemic steroids. Ocrelizumab was discontinued and therapy switched to ozanimod. **Figure 2** shows endoscopic images of the ileum of patient #9. At last follow-up at 28 months, the patient had incomplete improvement of gastrointestinal symptoms.

**Figure 3:**
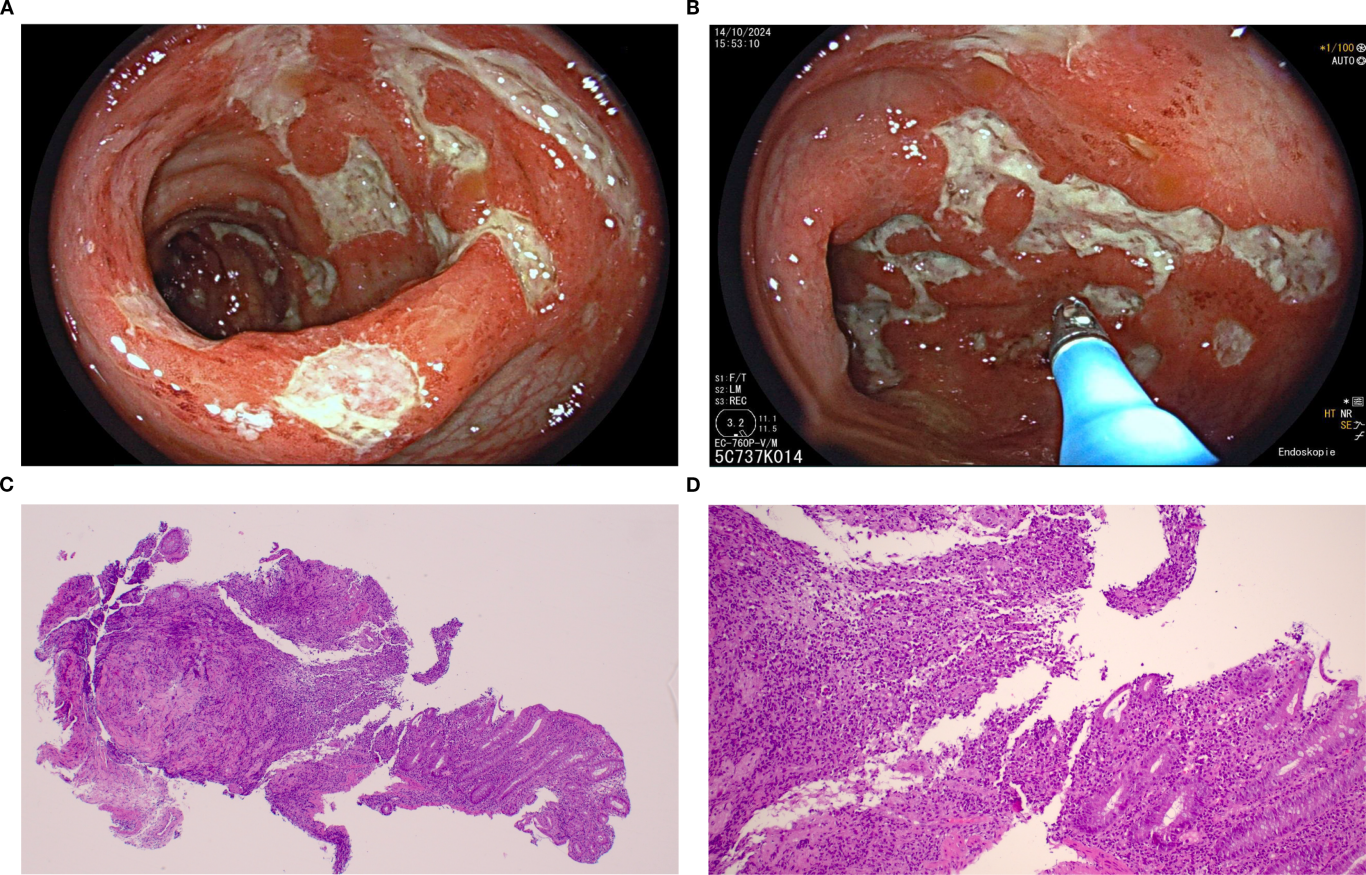
Pictures **(A)** and **(B)** show endoscopic appearance of mucosal injury in the colon of a patient (patient #10) with toxic colitis after seven infusions of ocrelizumab, as seen during colonoscopy. There are multiple deep ulcerations of different sizes and shapes which are partially confluent and surrounded by erythematous mucosa. The distribution is patchy throughout the whole colon, with intervening areas of completely normal mucosa. The endoscopic appearance is similar to Crohn’s colitis. Pictures **(C)** and **(D)** depict HE stained colon ascendens biopsies of the same patient with erosions, ulcers and apoptosis with minimal regenerative mucosal remodeling.

Endoscopic biopsy demonstrated toxic colitis in a 47-year-old patient (patient #10). **Figure 3** demonstrates the endoscopic and histological appearance of mucosal injury in the colon of patient #10. The patient was treated with systemic steroids and ocrelizumab was discontinued. At last follow-up at 9 months, she showed marked, albeit incomplete, improvement of gastrointestinal symptoms and endoscopic findings.

Another patient (patient #13), who had received the highest cumulative ocrelizumab dose (7200 mg) of all patients included in this work, developed biopsy proven lymphocytic colitis. Despite systemic steroid treatment and cessation of ocrelizumab, this patient showed no clinical improvement of gastrointestinal symptoms at last follow-up at 12 months.

#### 3.3.2 Acute gastroenterological adverse reactions

Two gastroenterological adverse reactions also occurred within a very short treatment interval following ocrelizumab therapy. One patient (patient #12) developed cholecystitis one week after the first dose of 300 mg ocrelizumab and a second patient (patient #11) had a perforated appendicitis two weeks after treatment with the second infusion of 300 mg ocrelizumab (cumulative dose 600 mg ocrelizumab). Both patients underwent surgery, completely recovered, and ocrelizumab therapy was continued without any further gastrointestinal events so far.

#### 3.3.3 Comparison of patients with dermatological and gastrointestinal adverse reactions

Patients with dermatological and gastrointestinal adverse reactions under ocrelizumab therapy did not differ regarding age at complication onset (*p* = 0.7), cumulative ocrelizumab dose (*p* = 1), interval between the start of ocrelizumab therapy and the onset of adverse reactions (*p* = 0.9), number of ocrelizumab infusions (*p* = 1), gender (*p* = 0.2) or previous treatment with DMTs (*p*>0.99).

## 4 Discussion

The key findings of this retrospective study on dermatological and gastrointestinal adverse reactions in a large single center cohort of ocrelizumab treated pwMS are i) the most frequent dermatological and gastrointestinal adverse reactions were various forms of psoriasis and inflammatory bowel diseases, ii) dermatological and gastrointestinal disorders can develop up to 6 years after the start of ocrelizumab therapy, iii) the spectrum of dermatological adverse reactions in ocrelizumab treated pwMS includes lichen planus, iv) we observed acute gastrointestinal complications following administration of ocrelizumab, v) while dermatological and gastroenterological treatments and adaptation of anti-CD20 therapy was associated with incomplete or complete improvement in approximately 75% of patients, 25% showed no improvement al last follow-up.

The cumulative frequencies of dermatological (1.8%) and gastrointestinal (1.1%) disorders developing under treatment with ocrelizumab observed in this work have to be regarded with caution as some pwMS were referred to our clinic because of these adverse events, which might have resulted in an increased frequency due to referral bias. To the best of our knowledge, systematic data from prospective observational studies on the incidence of dermatological and gastrointestinal adverse events in ocrelizumab treated pwMS are currently not available. Nevertheless, the frequencies of dermatological and gastrointestinal adverse reactions observed in this study suggest that clinicians treating pwMS with anti-CD20 therapies in daily clinical practice should be aware of these adverse reactions and should actively inquire about dermatological or gastrointestinal abnormalities. Furthermore, the risk for these adverse events should be discussed with patients during the informed consent process prior to initiating anti-CD20 treatment.

Concerning the question of a causal association of ocrelizumab and the observed dermatological and gastrointestinal disorders, our present findings are in line with previous reports of psoriasis and inflammatory bowel disease occurring in temporal association with anti-CD20 therapies, suggesting a causal contribution of ocrelizumab in the development of these disorders (8–11, 15). Furthermore, improvement of dermatological and gastrointestinal disorders in some of our patients after ocrelizumab cessation is likewise compatible with a causal role of anti-CD20 therapies.

Immune-mediated adverse reactions such as psoriasis, Graves’ disease and rheumatoid arthritis have also been reported with other DMTs used in MS, including interferons, natalizumab and alemtuzumab (16–20). Among the patients with ocrelizumab therapy and dermatological and gastrointestinal complications reported herein, 9/13 (69%) had previously been treated with various DMTs. While a possible contribution of those previous DMTs to the development of dermatological and gastrointestinal adverse reactions cannot be ruled out, development of dermatological and gastrointestinal disorders in 4 patients exclusively treated with anti-CD20 therapy supports a genuine role of anti-CD20 therapy.

An important finding of this study is that dermatological (nail psoriasis) and gastrointestinal adverse reactions (lymphocytic colitis) may develop up to 6 years following initiation of ocrelizumab therapy. To date, to the best of our knowledge, only one case report has described colitis in a pwMS following five years of ocrelizumab therapy (21). Interestingly, in the pivotal clinical trials of ocrelizumab, psoriasis and inflammatory bowel disease were not reported as adverse events during the observation period of 96 months. In a pooled analyses of the OPERA I and II trials, however, three cases of appendicitis were documented among participants treated with ocrelizumab, though three cases were also reported in the control group treated with interferons (1, 2). Overall, our present observations underscore that rare and long-term adverse reactions may be unrecognized during the typical timeframe of phase 3 clinical trials, highlighting the need for continuous pharmacovigilance following the approval of new drugs. Physicians treating pwMS with anti-CD20 therapies should be aware that adverse reactions may only occur after several years of treatment. While the optimum duration of anti-CD20 is currently unknown, many patients are presently treated beyond the 96-months duration of the pivotal clinical trials. The possibility of long-term adverse reactions should thus be considered when making decisions about continuation or adaptation (e.g. extending treatment intervals) in individual pwMS treated with anti-CD20 therapies.

Whereas, in keeping with previous reports, the most common dermatological adverse reactions were various forms of psoriasis (9, 19, 22), a rather novel finding is, that two patients developed lichen planus under anti-CD20 therapy. While one of these patients (patient #3) was previously reported in detail (14), patient #4 is a novel patient. Together with another previously reported pwMS with oral lichen planus (23), to the best of our knowledge, only three pwMS with lichen planus under ocrelizumab therapy have thus far been reported. In addition, one case report described a reactivation of a pre-existing lichen planus in a patient under ocrelizumab therapy (24), and at least two cases of lichen planus under rituximab in a patient with non-Hodgkin B-cell lymphoma and another patient with pemphigus vulgaris have previously been reported (25, 26). Of note, both of our patients with lichen planus showed no improvement despite dermatological treatment. Altogether, the previously reported and our present findings suggest that lichen planus may represent a rare and potentially difficult to treat adverse reaction from anti-CD20 therapy.

As a previously unreported possible dermatological adverse reaction, we observed one patient (patient #7) with rosacea. Rosacea is a common multifactorial dermatological disease with a strong genetic background (27). Altogether, a role of ocrelizumab in the development of rosacea in patient #7 can currently neither be proven nor excluded.

While alopecia has previously been described as an adverse reaction to anti-CD20 treatment (28, 29), we did not observe alopecia in our cohort. An analysis of FDA data identified 8,759 cases of alopecia among 44,114 reported adverse dermatological events in pwMS receiving various DMTs. 243 of these 8,759 (2.8%) cases of alopecia were associated with ocrelizumab (28). A case series describing five patients who developed alopecia within four month of ocrelizumab treatment noted full recovery with topical treatment (29).

Our data demonstrate that anti-CD20 therapy may be associated with short- and long-term adverse reactions in the gastrointestinal tract. Quesada-Simo et al. recently described a possible affection of all segments of the bowel, in patients receiving anti-CD20 therapy, with a predominance in the terminal ileum (10). Furthermore, the authors proposed diagnostic criteria for drug related IBD to guide physicians. In line with our observations, arguments in support of a causal association between the drug and the adverse reactions proposed by Quesada-Simo et al. include a good response to glucocorticoid treatment as well as clinical and endoscopic recovery following drug withdrawal (10).

We observed two patients with a short latency period of one and two weeks between the initiation of ocrelizumab treatment and the occurrence of perforated appendicitis and cholecystitis, respectively. Comparable short-term onset patterns have been described for hepatitis, acute diverticulitis and esophagitis during anti-CD20 treatment (30–32). Speculatively, such short-term adverse reactions might be linked to immediate immunological effects of anti-CD20 therapy, such as the loss of intestinal immunoglobulin A (IgA) and intestinal T cell dysregulation (31). However, since premedication with corticosteroids is mandatory directly before ocrelizumab treatment, a role of corticosteroids in the development of appendicitis and cholecystitis cannot be excluded.

Concerning the management of pwMS with dermatological or gastrointestinal complications under anti-CD20 therapy, treatment decisions need to take into account the severity of adverse symptoms, their response to disease-specific therapy as well as the current extent of MS disease activity. While stopping anti-CD20 therapy is an obvious option, in approximately half of our patients with adverse reactions under anti-CD20 therapy, treatment with ocrelizumab was continued to assure continuous control of MS. Of note, in these instances, extended-interval dosing (EID) with ocrelizumab infusions administered at longer than the standard 6-months intervals might be a reasonable therapeutic strategy, which we pursued in two patients with dermatological adverse reactions. This strategy is supported by studies showing low to no MS disease activity recurrence during therapy-free intervals of >12 months after ocrelizumab treatment cessation (33, 34). Frequent monitoring of pwMS after anti-CD20 therapy cessation or undergoing EID might be considered, including clinical examinations, MRI and determination of neurofilament light chain in serum. A switch of anti-CD20 therapies to alternative immunotherapies, which are effective against MS and the concomitant dermatological and gastrointestinal diseases, might also be considered. For example, fumaric acid esters are used in the therapy of MS and psoriasis (35). Likewise, natalizumab and ozanimod have been shown to reduce disease activity in both, MS and IBD (36, 37). For an in-depth review of therapeutic strategies for pwMS and concomitant psoriasis or IBD we also refer to the work of Brummer et al. (38). Altogether, pwMS and dermatological and gastrointestinal adverse reactions associated with anti-CD20 therapies need to be managed on an individual basis and ideally in close collaboration with treating dermatologists and gastroenterologists.

The precise mechanisms by which anti-CD20 therapy induces dermatological and gastrointestinal disorders remain unknown. As such disorders were observed only in a small proportion of pwMS receiving anti-CD20 treatment, we speculate that genetic factors and/or the sequence of other DMTs prior to the start of anti-CD20 treatment could contribute to immune dysregulation, ultimately resulting in inflammatory dermatological and gastrointestinal disorders.

One potential component in this immune dysregulation might be an overweight of proinflammatory factors. IBD and psoriasis share immunological similarities, as both diseases are primarily T-cell mediated. Pathophysiologically, an increased number of dysfunctional Interleukin-17 (IL-17) producing T-helper 17 cells (Th17), along with IL-12 and IL-23, play a central role in IBD and psoriasis (10). Regarding the association between IBD and psoriasis and anti-CD20 therapy, one hypothesis suggests that a loss of regulatory B cells and IL-10, combined with an overweight of the proinflammatory Th17 pathway, promotes the occurrence of psoriasis and IBD (8, 11, 19). Furthermore, in mouse models, rituximab has been shown to increase Th1 cells and proinflammatory cytokines such as IL-6 and tumor-necrosis-factor-α (TNF-α). In humans, B-cell depletion has likewise been associated with impaired B- and T-cell interaction, resulting in elevated Th1 and Th17 cell counts (10).

While data on concomitant autoimmune diseases in pwMS is somewhat heterogeneous (39), psoriasis, in particular, has been reported as co-occurring alongside MS. Whereas the prevalence of psoriasis in the general Western European population is reported to be 1.52% (40), studies of MS cohorts have described a prevalence ranging from 0.39% to 7.74% (42). A Canadian study reported a crude prevalence of psoriasis of 4666.1 per 100,000 persons in an MS population as compared to 3313.5 per 100,000 persons in a matched control population and a 54% higher risk of incident psoriasis in the MS population (41).

IBD prevalence in the general European population is estimated at about 0.35%, whereas in the MS population, reported prevalence ranges from 0.36% to 4.66% (42, 43). Notably, the development of autoimmune diseases is thought to be driven by shared risk factors. For example, IBD and MS share similarities environmental risk factors such as vitamin D deficiency, smoking and high socioeconomic status, as well as genetic variants (15). Furthermore, a meta-analysis demonstrated that in MS and IBD the pooled risk ratio for developing the other disease is increased by 50% (44). Although the frequencies of psoriasis and IBD occurring under ocrelizumab therapy observed in our cohort were thus in the range of that reported in general MS populations, this does not exclude a causative role of ocrelizumab therapy in the development of psoriasis and IBD. Rather, pwMS could have an intrinsically increased liability to develop psoriasis or IBD, which may individually contribute to the development of these diseases under anti-CD20 therapy.

Limitations of this study include its retrospective design and the variable follow-up intervals of the patients who developed dermatological and gastrointestinal adverse reactions. We thus cannot exclude that some of the dermatological and gastrointestinal adverse reactions could have improved or recurred after longer follow-up intervals. Furthermore, dermatological and gastrointestinal adverse reactions were identified by asking patients about potential adverse reactions of anti-CD20 therapy, but were not systematically assessed, e.g. by regular dermatological screenings. Therefore, it is possible that milder adverse reactions could have gone unnoticed. Finally, our cohort included pwMS treated with ocrelizumab, but no other currently approved anti-CD20 therapies. Nevertheless, we consider it likely that the findings observed in this study represent a class effect of anti-CD20 therapies, not confined to ocrelizumab. Indeed, two patients with psoriasis and one patient with colitis under treatment with ofatumumab have previously been reported (11, 22, 45). We are also aware of one patient with a positive family history of psoriasis who developed psoriasis vulgaris after two years of ofatumumab treatment (CO, personal observation). Likewise, psoriasis and colitis have also been documented with rituximab (9, 15, 20).

In conclusion, this study emphasizes that physicians involved in the care of pwMS should be aware of the broadening spectrum of dermatological and gastrointestinal adverse reactions under treatment with ocrelizumab/anti-CD20 therapies in pwMS, which may occur from weeks up to six years after initiation of ocrelizumab therapy. While dermatological and gastrointestinal treatments and adaptation of anti-CD20 therapy was associated with partial or complete improvement of symptoms, some patients showed no improvement at last follow-up. Future studies may address the questions of individual factors predicting the risk of dermatological and gastrointestinal adverse reactions and of the best strategies for adaptation of anti-CD20 therapies once dermatological and gastrointestinal complications have developed.

## Data Availability

The raw data supporting the conclusions of this article will be made available by the authors, without undue reservation.
